# Cataract services for all: Strategies for equitable access from a global modified Delphi process

**DOI:** 10.1371/journal.pgph.0000631

**Published:** 2023-02-22

**Authors:** Jacqueline Ramke, Juan Carlos Silva, Michael Gichangi, Thulasiraj Ravilla, Helen Burn, John C. Buchan, Vivian Welch, Clare E. Gilbert, Matthew J. Burton, The Cataract Access Study Group

**Affiliations:** 1 International Centre for Eye Health, London School of Hygiene and Tropical Medicine, London, United Kingdom; 2 School of Optometry and Vision Science, University of Auckland, Auckland, New Zealand; 3 Pan American Health Organization, World Health Organization, Bogotá, Colombia; 4 Ophthalmic Services Unit, Ministry of Health, Nairobi, Kenya; 5 Aravind Eye Care System, Madurai, India; 6 Bruyère Research Institute, University of Ottawa, Ottawa, Canada; 7 Moorfields Eye Hospital, London, United Kingdom; PLOS: Public Library of Science, UNITED STATES

## Abstract

Vision loss from cataract is unequally distributed, and there is very little evidence on how to overcome this inequity. This project aimed to engage multiple stakeholder groups to identify and prioritise (1) delivery strategies that improve access to cataract services for under-served groups and (2) population groups to target with these strategies across world regions. We recruited panellists knowledgeable about cataract services from eight world regions to complete a two-round online modified Delphi process. In Round 1, panellists answered open-ended questions about strategies to improve access to screening and surgery for cataract, and which population groups to target with these strategies. In Round 2, panellists ranked the strategies and groups to arrive at the final lists regionally and globally. 183 people completed both rounds (46% women). In total, 22 distinct population groups were identified. At the global level the priority groups for improving access to cataract services were people in rural/remote areas, with low socioeconomic status and low social support. South Asia and Sub-Saharan Africa were the only regions in which panellists ranked women in the top 5 priority groups. Panellists identified 16 and 19 discreet strategies to improve access to screening and surgical services, respectively. These mostly addressed health system/supply side factors, including policy, human resources, financing and service delivery. We believe these results can serve eye health decision-makers, researchers and funders as a starting point for coordinated action to improve access to cataract services, particularly among population groups who have historically been left behind.

## Introduction

Cataract is the leading cause of blindness globally, and a major cause of moderate and severe vision impairment, affecting an estimated 94 million people in 2020 [1]. Vision loss from cataract is unequally distributed between and within countries [1, 2]. Reducing cataract blindness and vision impairment was, therefore, a priority in the *Universal Eye Health*: *Global Action Plan 2014–2019* endorsed at the 66th World Health Assembly in 2013 [3]. More recently, strengthening cataract services and improving access to eye care was a key theme in the World Health Organization’s (WHO) first *World Report on Vision* in 2019 [4], and all countries committed to increasing effective cataract surgical coverage(eCSC) [5] at the World Health Assembly in 2021 [6].

This priority afforded to cataract services at the policy level over the last few decades likely contributed to the increase in the number of surgeries performed and the corresponding 32.7% reduction in the age standardized prevalence of cataract blindness between 1990 and 2020 (95% uncertainty interval 31·2–34.2%) [1]. However, population ageing and growth mean that despite the falling prevalence of cataract blindness, the number of people affected is likely to continue increasing [7]. In addition, insufficient monitoring of inequality in population-based surveys and lack of facility-based service data means we have little idea of the social distribution of recent gains [2, 8].

In the *World Report on Vision*, WHO positioned eye health as an essential component of Universal Health Coverage, and called for the use of evidence when planning eye care services [3]. Unfortunately, very little relevant evidence is available on how to realise universal eye health, including how to deliver cataract services to address inequity [2, 9]. For example, a Cochrane systematic review of interventions to improve access to cataract services in low- and middle-income countries (LMICs) found only two studies, both from China [10]. This evidence gap means resources may currently being wasted on ineffective strategies, and opportunities are being missed to scale-up effective strategies. This evidence gap was recognised in the Grand Challenges in global eye health process undertaken for the Lancet Commission, where 336 panellists from 118 countries ranked improving access to and quality of cataract services as the second highest priority for action in the field.

Over the past two decades many strategies have been implemented to strengthen cataract services in LMICs. While these have not been rigorously evaluated, service providers, decision-makers and their implementing partners (such as NGOs and health researchers) have gained knowledge and experience about the effectiveness, appropriateness and feasibility of these strategies. In this project we aimed to draw on this knowledge and experience through engaging multiple stakeholder groups in a Delphi process to prioritise (1) delivery strategies that improve access to cataract services for under-served groups and (2) population groups to target with these strategies across world regions.

## Methods

This study is reported according to the relevant items in the STROBE guideline [11], as well as Delphi specific guidance [12]. Ethics approval for this study was received from the London School of Hygiene & Tropical Medicine Ethics Committee (Ref 15824); all panellists provided written informed consent to participate and the study conformed to the principles embodied in the Declaration of Helsinki. The project was completed as part of the *Lancet Global Health* Commission on Global Eye Health [13].

### Panellist selection

A purposive sampling technique was employed to recruit a panel of stakeholders knowledgeable about delivery strategies for cataract services. We categorised panellists geographically using the seven Global Burden of Disease (GBD) Super Regions (Central Europe, Eastern Europe and Central Asia; High-income; Latin America and Caribbean; North Africa and Middle East; South Asia; Southeast Asia, East Asia and Oceania; Sub-Saharan Africa). To identify strategies for Small Island Countries which have very different health services context than much larger countries in their Super Region (e.g. Fiji and China, or Jamaica and Brazil), we analysed results separately for Small Island Countries, being countries within Oceania (removed from the Southeast Asia, East Asia & Oceania Super Region) and Caribbean (removed from the Latin America & Caribbean Super Region). We aimed to recruit 20–30 panellists in each region and from a range of stakeholder groups to allow different perspectives to be incorporated i.e. providers, decision-makers, and implementing partners.

The research team nominated stakeholders working in each of the regions and further nominations were sought from Lancet Commissioners. Nominated individuals were invited to participate in the two-round online process following informed consent. Panellists who completed both rounds were invited to join the study authorship group.

### Data collection

**Fig 1:**
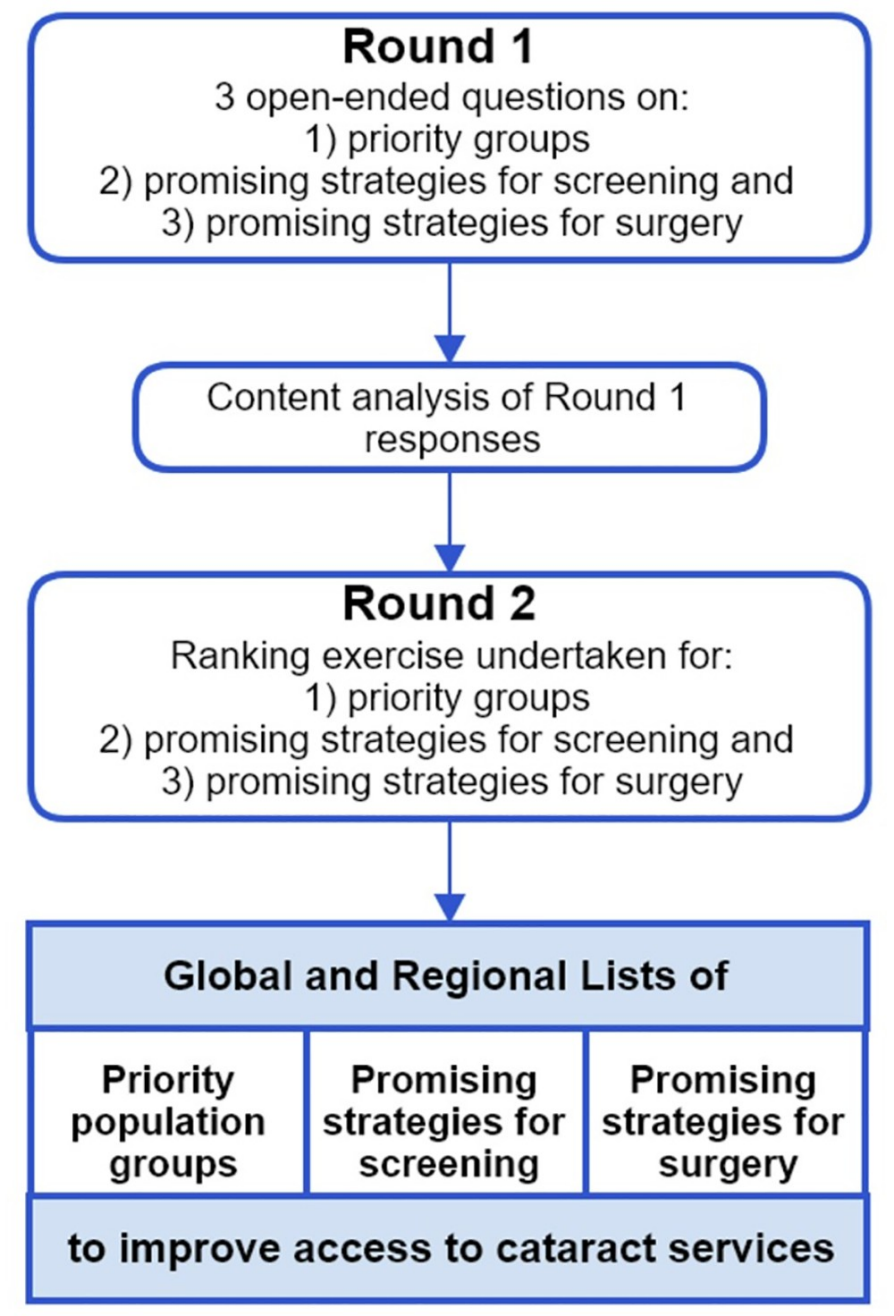
Summary of the Delphi process to prioritise strategies to reduce inequity of cataract vision impairment.

Data were collected in two online rounds (summarised in Fig 1) using Qualtrics (Qualtrics, 2019; Utah, USA, available at https://www.qualtrics.com). Prior to both rounds, members of the research team pilot-tested the online form for clarity and user experience, and modifications were incorporated. Both rounds were available in English, Spanish and French.

In Round 1, panellists provided information about their gender, country of employment, main stakeholder group and their eye care experience (<5, 5–9, 10–19 or 20+ years).

Panellists were then asked three open-ended questions for their setting (Box 1).

**Box 1. Questions in round 1.**

1. Which population subgroups experience the most difficulty accessing cataract services (screening and/or surgery)?
2. For people in the most under-served population groups and who have vision impairment from cataract, what interventions are effective at increasing their use of cataract screening services? (i.e. services where people with operable cataract are identified)
3. For people in the most under-served population groups and who have been identified with operable cataract, what interventions are effective at increasing their use of cataract surgical services? (i.e. they undergo surgery)

Panellists were encouraged to include as many opinions as possible to maximise the chance of identifying the most important ideas.

All ideas generated in round 1 were included in round 2, and no items were added by the research team during the analysis phase. The nominated population groups were de-duplicated and otherwise all were presented to panellists in round 2. For the nominated interventions, content analysis was used to identify the major themes for screening and surgery separately. This was undertaken by one researcher (JR) and verified by a further two (HB and MJB) to ensure all the original suggestions had been sufficiently captured.

In round 2, panellists were presented with all suggested population groups, which they ranked in two ways based on their experience in their country/region. First, they indicated on a five-point scale how much difficulty each group had accessing cataract services when they needed it, from the least/no difficulty (0) to the most/extreme difficulty (5). Second, they indicated which group represented the largest number of people experiencing difficulty accessing services when they needed it, from a very low number/none (0) to a very large number (5). For each group a ‘not applicable’ item was available if the panellist felt a group was not relevant in their setting (e.g. caste).

To rank the interventions, the same process was followed for screening and surgery separately, drawing on a method previously used [14]. Panellists were presented the list of nominated interventions and asked twelve questions (Box 2). For each question, panellists selected three to five strategies they thought the most promising in their context. After nominating strategies for all 12 questions, panellists were provided a summary of how many times they had selected each strategy in the preceding questions, and were then asked to rank their top eight strategies (1 = highest) to improve access to screening/surgery for cataract for under-served groups.

**Box 2. Questions presented for interventions in round 2.**

**Reach**: Which strategy would reach the most people within a community?

**Reach**: Which strategy would reach the people who need it most within a community?

**Acceptability**: Which strategy would be most acceptable to people with cataract and their families?

**Acceptability**: Which strategy would be most acceptable to health workers?

**Acceptability**: Which strategy would be most acceptable to the Ministry of Health?

**Equity**: Which strategy would improve screening uptake by women?

**Equity**: Which strategy would improve screening uptake by people of low socio-economic status?

**Equity**: Which strategy would improve screening uptake by people living in rural & remote areas?

**Feasibility**: Which strategy would be most feasible to implement for health services in the short term?

**Feasibility**: Which strategy would be most feasible to implement for health services in the long term?

**Cost**: Which strategy represents the best value for money in reaching underserved populations in the short term?

**Cost**: Which strategy represents the best value for money in reaching underserved populations in the long term?

### Analysis

For the priority groups, the total score for the two criteria was calculated for each region as well as globally and standardized to the number of panellists. In this way, the scores presented were between 0 and 5, with a higher score meaning the group was considered to 1) have more difficulty and 2) be a large group respectively.

To analyse the strategies, the top strategy for each panellist was allocated 8 points, their 2^nd^ ranked strategy 7 points and so on down to their 8^th^ strategy receiving 1 point; the remaining strategies (not in the top 8) were allocated no points. A total score was calculated for each strategy and standardized to the number of panellists. The final score was between 0 and 8, with a higher score reflecting a strategy considered more promising. Results were summarised using heat maps, and were also mapped to the patient-centred access framework developed by Levesque and colleagues [15].

## Results

We recruited 183 panellists who completed the two rounds, including 84 women (46%). The participation rate across the two rounds was 84%. The number of panellists was largest in Latin American and Small Island countries (both n = 26) and lowest in Central Europe, Eastern Europe & Central Asia (n = 16). The majority nominated their main field of work as service providers (n = 75, 41%) with similar numbers of researchers (n = 34, 19%), NGO implementers (n = 32, 17%) and Ministry of Health planners (n = 31, 17%). More than half (52%) had worked in eye care for at least 20 years (Table 1).

**Table 1:** Characteristics of panellists completing both rounds of the Delphi process.

Panellist characteristics		Completing both rounds n (%)
Sex	Female	84 (46)
	Male	99 (54)
Region*	Small Islands of Oceania & Caribbean**	26 (14)
	Latin America	26 (14)
	Sub-Saharan Africa	25 (14)
	South Asia	24 (13)
	High Income Countries	24 (13)
	Southeast Asia & East Asia	22 (12)
	North Africa & Middle East	20 (11)
	Central Europe, Eastern Europe & Central Asia	16 (9)
Main field of work	Service provider	75 (41)
	Researcher	34 (19)
	NGO	32 (17)
	Ministry of Health / planner	31 (17)
	Other (including educator)	11 (6)
Years working in eye care	<5	13 (7)
	5–9	24 (13)
	10–19	50 (27)
	20+	96 (52)
Total		183

*List of countries available in S1 Text;

**Oceania and Caribbean presented separately to their GBD Super-Region due to unique health service challenges in these settings

### Groups to prioritise

At the global level, rural/remote dwellers, people with low socioeconomic status and people with low social support were ranked in the top five in terms of having the most difficulty accessing services and being among the largest groups (Table 2). In terms of groups experiencing the most difficulty accessing services, the other two groups in the top five were people without housing and people with other disabilities. The other two groups ranked in the top five in terms of being a large group were the elderly and people with low education. Women were the 12^th^ ranked group globally; South Asia and Sub-Saharan Africa were the only regions in which women were included in the top five groups (based on size of the group). Regional differences were evident, with relatively high scores for some groups, such as caste in South Asia, nomadic people in Sub-Saharan Africa and North Africa & Middle East, Indigenous people in Latin America and ethnic minorities in Southeast & East Asia (Table 2).

**Table 2:** Priority groups to target to improve access to cataract services by region and globally in terms of the group 1) having the most difficulty accessing care when needed and 2) being the largest group experiencing difficulty accessing care when needed (prioritised by 183 panellists, April-June 2020, arranged by average global score of the two criteria, red = most difficulty / biggest group).

Priority Groups	Central / Eastern Europe & Central Asia		High-income countries		Latin America		North Africa & Middle East		Small Island countries		Southeast Asia & East Asia		South Asia		Sub-Saharan Africa		Global	
	Most difficulty	Biggest group	Most difficulty	Biggest group	Most difficulty	Biggest group	Most difficulty	Biggest group	Most difficulty	Biggest group	Most difficulty	Biggest group	Most difficulty	Biggest group	Most difficulty	Biggest group	Most difficulty	Biggest group
Rural / remote	3.00	2.06	2.00	1.74	3.73	3.46	2.90	2.86	3.36	3.00	3.65	3.30	3.92	3.38	4.00	3.48	3.36	2.96
Low socioeconomic status	2.38	2.50	2.26	1.87	3.38	3.62	2.76	2.71	3.08	2.84	3.00	3.30	3.38	3.13	3.84	3.72	3.05	3.00
Low social support	2.44	1.81	2.04	1.83	2.85	2.00	2.52	2.33	2.80	2.60	3.70	2.96	3.50	2.75	4.04	2.88	3.02	2.42
Other disabilities	2.19	1.75	2.48	1.61	2.73	1.88	2.57	1.71	2.88	2.12	3.30	2.35	3.67	2.38	4.00	1.96	3.02	1.98
Elderly	2.06	2.38	1.35	2.04	2.27	2.50	1.62	2.10	2.68	2.56	2.13	2.91	2.42	2.63	3.12	3.40	2.23	2.58
Without housing	2.63	1.31	2.83	1.22	3.73	2.08	2.52	1.62	2.84	1.84	3.35	1.57	3.25	2.29	3.00	1.72	3.05	1.73
Low education / health literacy	1.81	1.69	1.83	1.13	3.15	2.85	2.05	2.00	2.44	2.40	2.43	2.70	2.46	2.38	3.16	2.84	2.46	2.29
Informal/no employment	1.81	1.69	1.78	1.26	2.88	2.69	2.52	2.38	2.36	2.20	2.57	2.61	2.38	2.33	3.12	2.40	2.46	2.22
Co-mordities	1.88	2.13	1.52	1.30	2.38	1.65	2.05	1.62	2.40	2.04	3.30	2.30	2.58	2.46	2.96	1.92	2.42	1.92
Institutionalised*	1.94	1.31	2.30	1.48	2.88	1.65	1.95	1.67	2.32	1.84	2.57	1.78	2.79	1.88	3.04	1.96	2.51	1.72
Without health insurance	1.44	1.06	1.83	1.30	3.12	2.81	1.81	2.05	1.56	1.48	1.87	1.65	1.58	1.83	2.64	2.44	2.02	1.87
Women	1.06	1.06	0.74	0.96	1.00	1.38	1.52	1.57	1.64	1.72	1.78	2.04	2.42	2.79	2.76	2.96	1.64	1.85
Urban slums	0.31	0.69	0.96	0.65	2.42	2.00	1.76	1.57	1.64	1.48	1.96	1.52	1.83	1.92	2.56	1.80	1.75	1.50
Children	1.06	1.13	0.61	0.30	1.58	1.42	1.29	1.33	1.64	1.12	1.70	1.65	1.92	1.92	2.20	1.64	1.53	1.33
Ethnic minority / migrants	1.13	0.31	2.00	0.91	2.38	1.15	1.10	0.62	0.88	0.40	2.70	1.43	1.54	1.29	1.72	0.88	1.71	0.90
Refugees / asylum seekers	0.75	0.38	1.70	0.74	1.50	0.81	1.90	1.43	0.96	0.32	1.83	1.26	2.00	1.29	2.24	1.36	1.64	0.96
Indigenous	0.19	0.25	1.43	1.09	3.31	2.12	0.29	0.10	0.80	1.00	1.96	1.39	1.17	1.21	0.80	0.48	1.32	1.01
Language**	0.50	0.31	2.04	1.57	1.81	1.00	1.14	0.81	0.92	0.72	1.87	1.17	0.96	0.71	1.08	0.64	1.32	0.89
Nomadic	0.38	0.25	0.57	0.17	0.58	0.38	2.33	1.10	0.12	0.20	1.30	0.65	2.21	1.46	2.52	1.72	1.27	0.76
Transgender	0.31	0.13	0.65	0.26	1.58	0.77	0.86	0.43	0.68	0.36	0.78	0.61	1.75	1.04	0.24	0.24	0.89	0.50
Religious minority	0.56	0.38	0.35	0.22	0.50	0.19	0.57	0.52	0.40	0.28	1.22	0.96	0.92	0.71	0.68	0.52	0.65	0.47
Caste	0.13	0.06	0.09	0.09	0.31	0.12	0.48	0.24	0.08	0.20	0.48	0.30	1.38	1.54	0.24	0.32	0.40	0.37

Most difficulty on a scale from 0 = least/no difficulty to 5 = extreme/most difficulty; Biggest group on scale from 0 = none/very few to 5 = most/large number.

*including in prison, in nursing homes;

**don’t speak dominant language.

### Strategies to improve access to screening services

Responses in round 1 regarding strategies to improve access to screening for cataract were de-duplicated and categorised into 16 separate strategies in Round 2 which showed substantial regional variation (Table 3). Once prioritised, policy development was considered the most promising strategy at the global level (score = 3.8), followed by strengthening detection and referral skills of primary-level staff (3.6) and establishing permanent services closer to the community level (3.2). This latter strategy was particularly popular in South Asia, obtaining the highest score of all responses (5.8), possibly due to the success of Vision Centres in this region. The next highest scoring strategies at the regional level were policy development in Latin America (4.7), outreach in small island countries and improving efficiency in high-income countries (both 4.4). The strategy of integration was sixth most promising at the global level (2.9), being most highly rated in high-income countries (4.0), Southeast & East Asia (3.7) and Latin America (3.5). People-centredness ranked equal 12^th^ overall, alongside the similar-themed community partnership strategy (both 1.6). Eliminating out-of-pocket costs for screening ranked equal 4^th^ overall (3.1), being highest in North Africa & Middle East and Sub-Saharan Africa (both 3.5).

**Table 3:** Heat map of the most promising strategies to improve access to screening for cataract (prioritised by 183 panellists, April-June 2020, arranged by global score).

Strategies to improve access to screening for cataract	Central / Eastern Europe & Central Asia	High-income countries	Latin America	North Africa & Middle East	Small Island countries	Southeast Asia & East Asia	South Asia	Sub-Saharan Africa	Global
Establish a primary eye care / screening program through national **policies, guidelines, budgets and plans**	3.4	3.5	4.7	4.2	4.0	4.0	3.1	3.3	3.8
Strengthen **skills of staff at primary level** (GPs, nurses, primary health workers, optometrists as relevant) to screen VA, detect cataract and refer in line with treatment guidelines [provide supportive supervision with effective follow-up care]	3.8	3.3	4.2	2.8	4.0	3.3	3.4	3.6	3.6
Establish **permanent primary eye services closer** to community level (e.g. vision centres, primary eye care centres)	2.6	2.2	2.9	2.6	2.8	2.4	5.8	3.8	3.2
**Eliminate out of pocket costs** for patients e.g. free screening, include eye screening in insurance coverage, provide transport, tiered pricing, cross-subsidy	2.6	3.0	2.8	3.5	3.0	3.0	3.3	3.5	3.1
Provide regular **outreach screening** (linked to surgical services) at community facilities to reduce the need for travel to a central facility	2.0	2.8	2.3	1.7	4.4	2.4	4.3	4.0	3.1
Improve **collaboration and integration** between levels of care, including referral (e.g. between primary and secondary care / between optometry and ophthalmology / between government, private & NGO sector)	3.1	4.0	3.5	3.0	2.3	3.7	2.2	1.9	2.9
**Raise awareness** (e..g. radio, someone who previously had surgery, women’s groups)—health education & promotion—eye problems and screening / treatment options / where services are available	2.2	1.7	2.2	4.2	3.2	3.1	2.3	2.9	2.7
Target screening to **under-served/at risk groups** e.g. nursing homes, people without housing, incarcerated, refugees, people with disability, those aged 65+, newborns	3.3	3.6	2.3	2.9	1.8	2.8	1.6	1.0	2.3
Improve **efficiency** of public outpatient clinics / reduce waiting times / minimise the number of visits required	2.4	4.4	2.0	1.9	2.3	1.3	0.6	1.8	2.1
Include screening in **established community-based activities** such as trachoma trichiasis, NCD screening, elderly program, newborn screening, or traditional health programs	2.6	0.8	0.5	2.1	2.2	2.6	1.7	2.2	1.8
Train **community case finders**; house to house screening, counselling and referral by community health workers	1.9	0.3	1.8	1.0	2.3	1.5	1.9	2.8	1.7
Strengthen **partnership with communities**—engage to provide culturally safe services / work with leaders to build trust / allow community control	1.1	1.6	1.6	1.4	1.3	1.8	1.8	2.1	1.6
Improve **people-centred care**—use accessible language during counselling; treat patients with humanity, quality, warmth; be trustworthy and transparent	2.6	1.7	2.5	1.4	0.7	1.2	1.2	1.4	1.6
Use **telemedicine** (and other technology such as AI) to improve access for remote communities	2.0	1.3	2.1	1.0	0.7	1.3	2.0	0.9	1.4
Screen at **community events** or services that target groups would attend e.g. health fairs, church events, licence renewal	0.1	0.7	0.2	1.5	1.1	0.5	0.6	0.9	0.7
Provide **accommodating services** (such as child minding, after-hours appointments) to enable participation in screening	0.4	1.1	0.5	0.7	0.0	1.1	0.1	0.0	0.5

Each panellist ranked their top 8 most promising strategies, a higher score reflects a strategy considered more promising.

The strategies selected by panellists as most promising for each of the criteria in Box 2 varied widely (S1 Fig). The final ranking of strategies reflected an emphasis on the criteria of *reach* as well as *long-term feasibility* and *long-term value for money*. The greatest consensus across the criteria was on eliminating out of pocket costs to improve access to screening for people of low socioeconomic status (selected by 127 panellists), strengthening skills as the most *acceptable* strategy for health workers (n = 124) and establishing policies, plans and budgets as the most *acceptable* strategy for the Ministry of Health (n = 124).

### Strategies to improve access to surgical services

A total of 19 separate strategies to improve access to surgical services were nominated in round 1 and prioritised in round 2 (Table 4). At the global level, the strategy considered most promising was decentralizing services so that surgical capacity was permanently available at the district or sub-district level (score = 3.6), followed by strengthening integration between screening, referral, surgery and follow-up (3.0), reducing/eliminating out-of-pocket costs for surgery and post-operative care (2.9), providing universal health insurance that includes cataract surgery (2.6) and delivering good quality outcomes (2.5). Making services more people-centred ranked 12^th^ overall.

**Table 4:** Heat map of the most promising strategies to improve access to surgery for cataract (prioritised by 183 panellists, April-June 2020, arranged by global score).

Strategies to improve access to cataract surgery	Central / Eastern Europe & Central Asia	High-income countries	Latin America	North Africa & Middle East	Small Island countries	Southeast Asia & East Asia	South Asia	Sub-Saharan Africa	Global
**Decentralize services** so services are closer to people–establish **permanent surgical capacity** at district/subdistrict level	2.8	3.1	3.7	4.1	3.8	2.5	4.4	4.2	3.6
Strengthen **integration between screening, referral to surgery and follow-up**	4.0	3.7	1.9	3.8	3.2	2.6	2.7	2.6	3.0
**Reduce or eliminate out of pocket costs** for surgery and post-operative care (e.g. have a subsidised option, philanthropy, insurance)	1.9	2.0	3.4	3.3	3.4	3.4	2.0	3.2	2.9
Provide **universal health insurance** that covers surgery and post-operative care	3.3	2.1	2.7	2.8	2.3	1.5	3.3	2.8	2.6
Deliver consistently **good quality surgical outcomes** to maximise uptake (monitor outcomes with audit and feedback and improve where necessary)	2.0	0.9	2.8	2.3	2.4	2.9	2.9	3.2	2.5
**Improve efficiency** to increase surgical output and reduce waiting time & unit cost e.g. day surgery, reduce number of appointments needed, adequate workforce, monitor productivity and link it to payment, twin-table theatres	3.0	4.3	3.2	1.0	1.9	2.4	0.7	2.4	2.3
Conduct **regular outreach surgery** away from the main eye department, linked to screening program	1.6	1.8	2.3	1.0	3.6	2.5	2.0	2.8	2.3
Design **services and funding mechanisms that give priority to under-served groups** e.g. target services to specific groups such as women in rural areas	2.1	3.9	1.7	2.3	1.6	2.8	1.2	1.6	2.1
**Raise awareness** through health education & promotion—benefits of timely surgery	1.8	1.2	1.2	2.4	2.2	1.7	2.5	2.3	1.9
Train more **surgeons in relevant surgical techniques** (e.g. MSICs) and **deploy / incentivize them** to work in rural areas / with under-served populations	1.6	0.7	3.1	1.9	2.8	1.7	1.5	1.5	1.9
**Assist with non-medical costs** such as accommodation, transport, escort support	1.3	2.5	0.9	2.2	1.6	2.1	2.2	1.0	1.7
Engage in **public-private-NGO partnerships** to maximise use of available resources (e.g. HR, facilities)	1.3	0.9	1.9	2.5	1.2	1.4	2.2	1.8	1.7
Make services more **culturally accessible and people-centred**; build trust by strengthening community relationships; involve local personnel; maintain continuity of service providers; reduce language and other barriers	1.8	2.7	2.0	1.4	0.6	1.9	0.9	1.7	1.6
Create strong **links with community health workers**—work with them to develop responsive services and support uptake of services and follow-up	1.1	1.2	1.6	0.6	2.0	1.5	2.4	1.9	1.6
**Provide transport** to and from services for the patient and their carer	2.1	1.4	1.2	1.5	1.5	1.7	1.8	1.2	1.5
Provide **counselling** to the patient and their family (by a nurse / peer) to discuss details of surgery, answer questions and secure social support for surgery	0.9	0.3	1.3	0.3	1.2	0.9	1.2	1.0	0.9
Improve **physical access** to services e.g. for people with other disabilities	1.8	1.2	0.2	1.3	0.4	0.7	0.6	0.4	0.8
Provide **more accommodating services**—evening/ weekend appointments, childcare, accept patients without a referral letter	0.8	1.9	0.7	0.9	0.3	0.9	0.4	0.2	0.7
Use **demand side financial schemes** such as vouchers or cash transfers to increase access and incentivise service providers to provide good quality services	1.0	0.3	0.3	0.3	0.0	1.0	1.1	0.2	0.5

Each panellist ranked their top 8 most promising strategies, a higher score reflects a strategy considered more promising.

As seen for screening strategies, there was regional variation in terms of promising strategies for surgery. For example, providing consistently good quality outcomes ranked 5^th^ at the global level (2.5), but was not a priority in high-income countries where outcomes tend to be routinely good (0.9). Instead, the most promising strategies in high-income countries—efficiency (4.3) and designing services and funding mechanisms that prioritise under-served groups (3.9)—were not among the top 5 strategies globally. Regular outreach surgery ranked most highly in small island countries (3.6).

As seen for screening, the strategies selected by panellists as most promising to improve access to surgery for each of the criteria in Box 2 varied widely, and again appeared to reflect an emphasis on the criteria of *reach* as well as *long-term feasibility* and *long-term value for money* (S2 Fig). Once again, the greatest consensus across the criteria was on reducing or eliminating out of pocket costs for people of low socioeconomic status (selected by 120 panellists), followed by decentralizing services which was considered the most *feasible long-term* strategy (n = 104) and the best *long-term value for money* (n = 102).

### Service accessibility dimensions

The identified strategies for each of screening and surgical services mapped across all stages of Levesque’s patient-centred access model [15] (S3 Fig). The service accessibility dimensions for which panellists nominated the most strategies for both screening and surgery were availability/accommodation (5 strategies for screening, 6 for surgery) and appropriateness (4 for screening, 5 for surgery). The prioritised strategies for screening covered the dimensions of availability/accommodation (n = 3), acceptability (n = 1) and affordability (n = 1) while prioritised strategies for surgery mapped to affordability (n = 2), appropriateness (n = 2) and availability /accommodation (n = 1).

## Discussion

We believe this is the first attempt to prioritise the most promising strategies to improve access to cataract services, as well as the population groups to target with these strategies. At the global level, the population groups prioritised by panellists were people living in rural/remote locations, with low socioeconomic status or with low social support, all of which have been documented to have differential access to cataract services in certain settings [16–19]. Panellists identified a broad range of strategies to improve access to cataract services that map across all stages of the Levesque access framework [15].

The variety of groups nominated by panellists and inter-regional differences highlight the need for context-specific monitoring approaches for cataract services, ideally in conjunction with all countries monitoring service access using a small number of core social variables—such as sex/gender, place of residence (urban/rural) and socioeconomic status—to allow global monitoring [20, 21]. For example, Indigenous peoples in Latin America, Nomadic peoples in Sub-Saharan Africa and North Africa & Middle-East and Caste in South Asia were prioritised while tending to not be highly prioritised in other regions.

In some contexts, support will be required to strengthen the health information system to enable monitoring of inequality and the subsequent use of the information in policy and planning. Plans are underway to expand the social variables collected in the most commonly implemented survey tool, the Rapid Assessment of Avoidable Blindness [22]. These additional population-based data will enable intermittent monitoring of access to effective cataract services among different population groups, and inform subsequent service targets. Expanding data collection at health facilities may be much more complex [21]. Further, collection of dimensions such as urban/rural is more straightforward than social support.

We were surprised that few regions prioritised women as a population group to target, leading to women not being in the top half of priority groups at the global level. This is despite evidence that women have lower access to cataract services in analyses at the global level [23, 24] and in the South Asia region [25], although not in Latin America [26]. Further, gender is the one social dimension for which there is evidence of a persistent inequality, with women having a higher prevalence of blindness due to cataract in GBD models in 2010, 2015 and 2020 [1, 27, 28]. One possible explanation for women not being prioritised despite these documented inequalities is that in any given context, women are not universally worse-off compared to men. For example, when data from national surveys of vision impairment and blindness in Nigeria and Sri Lanka were disaggregated by sex concurrently with urban/rural domicile and marital status, in both countries married women in urban centres had the lowest prevalence of cataract blindness of all groups, while women living in rural areas without a spouse had the highest [17]. Our unidimensional approach used here may have caused panellists to rank women differently than if they had been able to consider intersections of gender with other axes of social disadvantage. Indeed, several panellists noted this accumulation of disadvantage among women in their free text comments in round 2. Intersectionality has been infrequently explored in cataract services research, but the existing examples consistently show that women are disproportionately among the worst-off [17, 29]. We encourage researchers from more contexts to add to this evidence-base on intersectionality so that more nuanced strategies can be developed that target the population subgroups most in need.

The *World Report on Vision* and the *Lancet Global Health* Commission on global eye health both emphasize the importance of eye health to realising Universal Health Coverage [4, 13]. The strategies identified in this process promote the Universal Health Coverage dimensions of access, quality, equity and financial protection to a greater or lesser extent. Access is embedded in all strategies proposed, while panellists ranked financial protection the highest of the other dimensions, highlighting the prominence of cost as a barrier to cataract services [30, 31]. Integration and people-centredness were further concepts emphasized in the *World Report on Vision* and expanded on by the Lancet Commission. Panellists ranked integration quite highly as a strategy to improve access to both screening and surgery, reflecting the importance of effective referral processes between levels of care and practitioners. In contrast, strategies focused on people-centredness (/accommodation) tended to be ranked low except by panellists from high-income countries.

This and other regional differences observed in our results are understandable and reinforce the need to develop context-specific approaches to improve access to cataract services. For example, strategies to improve efficiency of screening and surgery were a priority in the High-Income region and was a low priority in most other regions, whereas panellists from High-Income countries gave a much lower priority to improving quality, reflecting the good results generally achieved [13]. A further example was Small Island Countries, which prioritised outreach services to a much greater extent than other regions, reflecting the very limited human resources often available in these countries. Within all regions, countries could review the list during their planning process, and determine which are the most promising and feasible in their setting. We believe most countries could identify one or more strategies to implement across the community-, facility-, system- or policy-level, recognising that a multi-faceted approach is likely to achieve the greatest gains.

Given the prominence of cataract as a priority in global initiatives over the past two decades, there should be more evidence on how to improve access to cataract services for all than what is available [2, 9, 32]. The reason for this evidence gap may be an assumption from eye care stakeholders that access will ‘trickle down’, whereby everyone benefits eventually as services expand and improve [33]. Australia provides evidence that this assumption is false—in the context of universally available, high quality services, a national survey in 2016 revealed that approximately 5 out of 10 Indigenous Australians who could benefit from cataract surgery had accessed surgery and obtained a good outcome, compared to almost 9 out of 10 non-Indigenous Australians [34]. An alternative explanation for the lack of evidence could be because cataract surgery is an efficacious intervention [35, 36], there has been less attention given to delivery strategies to reach under-served groups relative to other conditions with less efficacious treatment. For example, a recent scoping review of service delivery models to improve access to eye care for Indigenous peoples found very few reports focused on strengthening cataract services (4 of 67 reports), compared to many more focused on diabetic retinopathy (n = 30) [37].

Regardless of the reason for the current lack of evidence, in this era of the SDGs and Universal Health Coverage—and given the commitment all countries made to increase eCSC this decade [6]—we believe it is essential to generate the evidence required for decision-makers in different settings to provide equitable cataract services. The results of the study reported here can serve as a menu of strategies that can be tested, alone or in combination with others, perhaps targeting specific groups of people as the context indicates. Given the scale of the problem, a coordinated approach perhaps within a series of implementation laboratories with a particular focus on equity and intersectionality would be ideal [9, 38]. There are examples from other fields such as maternal and child health, where multi-country implementation research has tested multifaceted, integrated health system approaches to improve services along the care continuum [39].

Our results must be considered in the context of several limitations. First, due to logistical challenges given the global nature of this process, we did not recruit people with cataract and their families onto the panel. This omission could mean we have missed important strategies from the people who have experienced challenges accessing cataract services and may have contributed to the low priority given to patient-centred strategies. Second, our results reflect the views of the invited panellists, and the global and regional level results presented may miss important national and sub-national priorities. To overcome this issue, we propose our list is used as a guide, with more nuanced interpretation at the national and sub-national level based on the local context. Third, we recognise the importance of intersectionality and accumulation of disadvantage leading to worse eye health outcomes [29], and believe our unidimensional assessment of population groups may have led to de-prioritisation of women as discussed above. Finally, our data collection overlapped with the early stage of the Covid-19 pandemic, so the strategies identified do not take account of the additional and extensive challenges countries are now experiencing.

We believe these results can serve eye health decision-makers, researchers and funders as a starting point for coordinated action to improve access to cataract services, particularly among population groups who have historically been left behind. The implementation of one or more of these strategies with rigorous monitoring and evaluation and subsequent dissemination of findings—disaggregated by social axes—would enhance our understanding of what works to improve access to cataract services, for who, and in what circumstances. This knowledge is essential for us to reduce the prevalence and unequal distribution of vision loss from cataract, and ultimately realise universal health coverage.

## Supporting information

S1 Text List of countries of panellists (n = 86). (PDF)

S1 Fig Number of panellists selecting the delivery strategy to improve access to cataract screening services for each criterion presented in round 2. (PDF)

S2 Fig Number of panellists selecting the delivery strategy to improve access to cataract surgical services for each criterion presented in round 2. (PDF)

S3 Fig Promising strategies to improve access to screening and surgery for cataract mapped to Levesque et al.’s patient-centred access framework [15]. (PDF)

S1 Data Round 2 responses from panellists. (XLSX)

S1 Acknowledgments Members of The Cataract Access Study Group. (PDF)
