# Supplementary material for: Cataract services for all: Strategies for equitable access from a global modified Delphi process
Source: PLOS Glob Public Health. 2023 Feb 22;3(2):e0000631. doi: 10.1371/journal.pgph.0000631 (PMC10021896; doi:10.1371/journal.pgph.0000631)
Supplement: S1 Text — (PDF) [file pgph.0000631.s001.pdf]

S1 Text: List of countries of panellists (n=86)

Central Europe, Eastern Europe & Central Asia: Armenia, Bosnia and Herzegovina, Czech Republic, Hungary, Kyrgyzstan, Latvia, Mongolia, Poland, Republic of Moldova, Romania, Russian Federation, Tajikistan, Ukraine
High-income countries: Australia, Canada, Chile, Israel, Italy, Japan, Netherlands, New Zealand, Portugal, Singapore, Spain, United Kingdom, United States of America
Latin-America*: Bolivia, Brazil, Colombia, Costa Rica, Ecuador, Guatemala, Honduras, Mexico, Panama, Paraguay, Peru
North Africa & Middle East: Afghanistan, Egypt, Iran, Morocco, Oman, Palestine, Tunisia, Turkey, United Arab Emirates
South Asia: Bangladesh, Bhutan, India, Nepal, Pakistan
Southeast Asia & East Asia*: Cambodia, China, Indonesia, Malaysia, Philippines, Sri Lanka, Thailand, Timor-Leste, Viet Nam
Sub-Saharan Africa: Cameroon, Ethiopia, Ghana, Kenya, Mozambique, Nigeria, Rwanda, Swaziland, Tanzania, The Gambia, Togo, Uganda, Zambia

Small island countries of Oceania & Caribbean: Barbados, Belize, Fiji, Guyana, Haiti, Jamaica, Kiribati, Papua New Guinea, Saint Lucia, Samoa, Solomon Is, Tonga, Trinidad & Tobago

*Oceania and Caribbean presented separately from their GBD Super-Region due to unique health service challenges in these settings
