# Supplementary material for: Cataract services for all: Strategies for equitable access from a global modified Delphi process
Source: PLOS Glob Public Health. 2023 Feb 22;3(2):e0000631. doi: 10.1371/journal.pgph.0000631 (PMC10021896; doi:10.1371/journal.pgph.0000631)
Supplement: S1 Fig — (PDF) [file pgph.0000631.s002.pdf]

S1 Fig: Number of panellists selecting the delivery strategy to improve access to cataract screening services for each criterion presented in Round 2 (arranged in the order of prioritised strategies following Round 2)

Strategies to improve access to cataract screening	Reach		Acceptability			Equity*			Feasibility		Value for money		Total
	Most people	Most in need	People & families	Health workers	MoH	Women	SES	Rural	Short-term	Long-term	Short-term	Long-term	
Establish a primary eye care / screening program through national **policies, guidelines, budgets and plans**	96	71	31	77	124	25	40	36	41	108	35	99	783
Strengthen **skills of staff at primary level** (GPs, nurses, primary health workers, optometrists as relevant) to screen VA, detect cataract and refer in line with treatment guidelines [provide supportive supervision with effective follow-up care]	84	72	45	124	80	29	35	57	68	77	54	76	801
Establish **permanent primary eye services closer** to community level (e.g. vision centres, primary eye care centres)	83	62	63	70	62	55	75	78	33	85	39	85	790
**Eliminate out of pocket costs** for patients e.g. free screening, include eye screening in insurance coverage, provide transport, tiered pricing, cross-subsidy	70	76	100	29	22	63	127	69	36	58	63	53	766
Provide regular **outreach screening** (linked to surgical services) at community facilities to reduce the need for travel to a central facility	72	80	86	43	40	43	83	116	88	38	77	44	810
Improve **collaboration and integration** between levels of care, including referral (e.g. between primary and secondary care / between optometry and ophthalmology / between government, private & NGO sector)	75	54	44	102	111	19	24	36	52	88	46	76	727
**Raise awareness** (e..g. radio, someone who previously had surgery, women's groups) - health education & promotion - eye problems and screening / treatment options / where services are available	73	67	65	45	59	79	46	53	77	55	64	56	739
Target screening to **vulnerable/at risk groups** e.g. nursing homes, the homeless, incarcerated, refugees, people with disability, those aged 65+, newborns	61	79	44	23	27	37	82	24	68	25	82	37	589
Improve **efficiency** of public outpatient clinics / reduce waiting times / minimise the number of visits required	55	40	77	60	65	33	27	26	59	51	47	60	600
Include screening in **established community-based activities** such as trachoma trichiasis, NCD screening, elderly program, newborn screening, or traditional health programs	35	38	30	35	53	25	41	42	36	35	57	30	457
Train **community case finders**; house to house screening, counselling and referral by community health workers	31	54	43	37	20	40	47	67	38	26	48	33	484
Strengthen **partnership with communities** - engage to provide culturally safe services / work with leaders to build trust / allow community control	30	49	40	36	38	40	37	41	27	38	18	47	441
Improve **people-centred care** - use accessible language during counselling; treat patients with humanity, quality, warmth; be trustworthy and transparent	36	31	73	33	24	39	35	15	30	41	25	33	415
Use **telemedicine** (and other technology such as AI) to improve access for remote communities	34	28	12	48	43	18	20	59	36	61	32	46	437
Screen at **community events** or services that target groups would attend e.g. health fairs, church events, licence renewal	12	19	33	14	8	31	40	34	52	8	42	14	307
Provide **accommodating services** (such as child minding, after-hours appointments) to enable participation in screening	4	12	32	8	2	49	35	15	9	7	14	6	193

*Each equity question was only presented to panellists who identified the group as having access difficulty in the first question; the denominator is therefore lower than for other criterion, particularly for women.
