# Supplementary material for: Cataract services for all: Strategies for equitable access from a global modified Delphi process
Source: PLOS Glob Public Health. 2023 Feb 22;3(2):e0000631. doi: 10.1371/journal.pgph.0000631 (PMC10021896; doi:10.1371/journal.pgph.0000631)
Supplement: S2 Fig — (PDF) [file pgph.0000631.s003.pdf]

S2 Fig: Number of panellists selecting the delivery strategy to improve access to cataract surgical services for each criterion presented in Round 2 (arranged in the order of prioritised strategies following Round 2)

Strategies to improve access to cataract surgery	Reach		Acceptability			Equity*			Feasibility		Value for money		Total
	Most people	Most in need	People & families	Health workers	MoH	Women	SES	Rural	Short-term	Long-term	Short-term	Long-term	
**Decentralize services** so services are closer to people – establish **permanent surgical capacity** at district/subdistrict level	90	87	68	56	88	48	60	92	26	104	39	102	860
Strengthen **integration between screening, referral to surgery and follow-up**	64	44	32	80	85	24	37	47	77	64	61	48	663
**Reduce or eliminate out of pocket costs** for surgery and post-operative care (e.g. have a subsidised option, philanthropy, insurance)	70	77	92	33	16	60	120	58	37	29	66	36	694
Provide **universal health insurance** that covers surgery and post-operative care	72	54	60	36	66	38	79	37	23	69	32	88	654
Deliver consistently **good quality surgical outcomes** to maximise uptake (monitor outcomes with audit and feedback and improve where necessary)	69	42	70	77	56	22	25	24	45	75	41	61	607
**Improve efficiency** to increase surgical output and reduce waiting time & unit cost e.g. day surgery, reduce number of appointments needed, adequate workforce, monitor productivity and link it to payment, twin-table theatres	73	42	56	83	86	28	30	29	56	81	58	86	708
Conduct **regular outreach surgery** away from the main eye department, linked to screening program	56	49	26	38	33	26	37	74	59	28	60	26	512
Design **services and funding mechanisms that give priority to disadvantaged groups** e.g. target services to specific groups such as women in rural areas	33	64	27	24	38	50	63	33	34	38	41	42	487
**Raise awareness** through health education & promotion - benefits of timely surgery	55	40	28	35	56	54	26	33	66	35	46	37	511
Train more **surgeons in relevant surgical techniques** (e.g. MSICs) and **deploy / incentivize them** to work in rural areas / with disadvantaged populations	49	27	9	75	57	6	9	34	24	83	21	76	470
**Assist with non-medical costs** such as accommodation, transport, escort support	26	43	77	17	7	36	91	40	38	13	49	17	454
Engage in **public-private-NGO partnerships** to maximise use of available resources (e.g. HR, facilities)	46	37	8	41	87	12	26	24	54	47	54	52	488
Make services more **culturally accessible and people-centred**; build trust by strengthening community relationships; involve local personnel; maintain continuity of service providers; reduce language and other barriers	36	41	44	34	28	49	22	24	33	39	24	34	408
Create strong **links with community health workers** - work with them to develop responsive services and support uptake of services and follow-up	27	42	19	76	39	33	28	50	48	37	37	38	474
**Provide transport** to and from services for the patient and their carer	28	57	61	12	7	30	74	72	49	6	45	10	451
Provide **counselling** to the patient and their family (by a nurse / peer) to discuss details of surgery, answer questions and secure social support for surgery	22	23	68	28	9	53	17	11	45	14	28	9	327
Improve **physical access** to services e.g. for people with other disabilities	18	38	28	6	8	8	17	13	19	8	13	16	192
Provide **more accommodating services** - evening/ weekend appointments, childcare, accept patients without a referral letter	16	16	53	12	6	57	28	27	33	24	23	11	306
Use **demand side financial schemes** such as vouchers or cash transfers to increase access and incentivise service providers to provide good quality services	9	10	14	16	13	5	33	8	9	16	26	17	176

*Each equity question was only presented to panellists who identified the group as having access difficulty in the first question; the denominator is therefore lower than for other criterion, particularly for women.
