# Supplementary material for: Cataract services for all: Strategies for equitable access from a global modified Delphi process
Source: PLOS Glob Public Health. 2023 Feb 22;3(2):e0000631. doi: 10.1371/journal.pgph.0000631 (PMC10021896; doi:10.1371/journal.pgph.0000631)

S3 Fig: Promising strategies to improve access to screening and surgery for cataract mapped to Levesque et al.'s patient-centred access framework[13] (bold=top 5 global priority for screening and surgery)

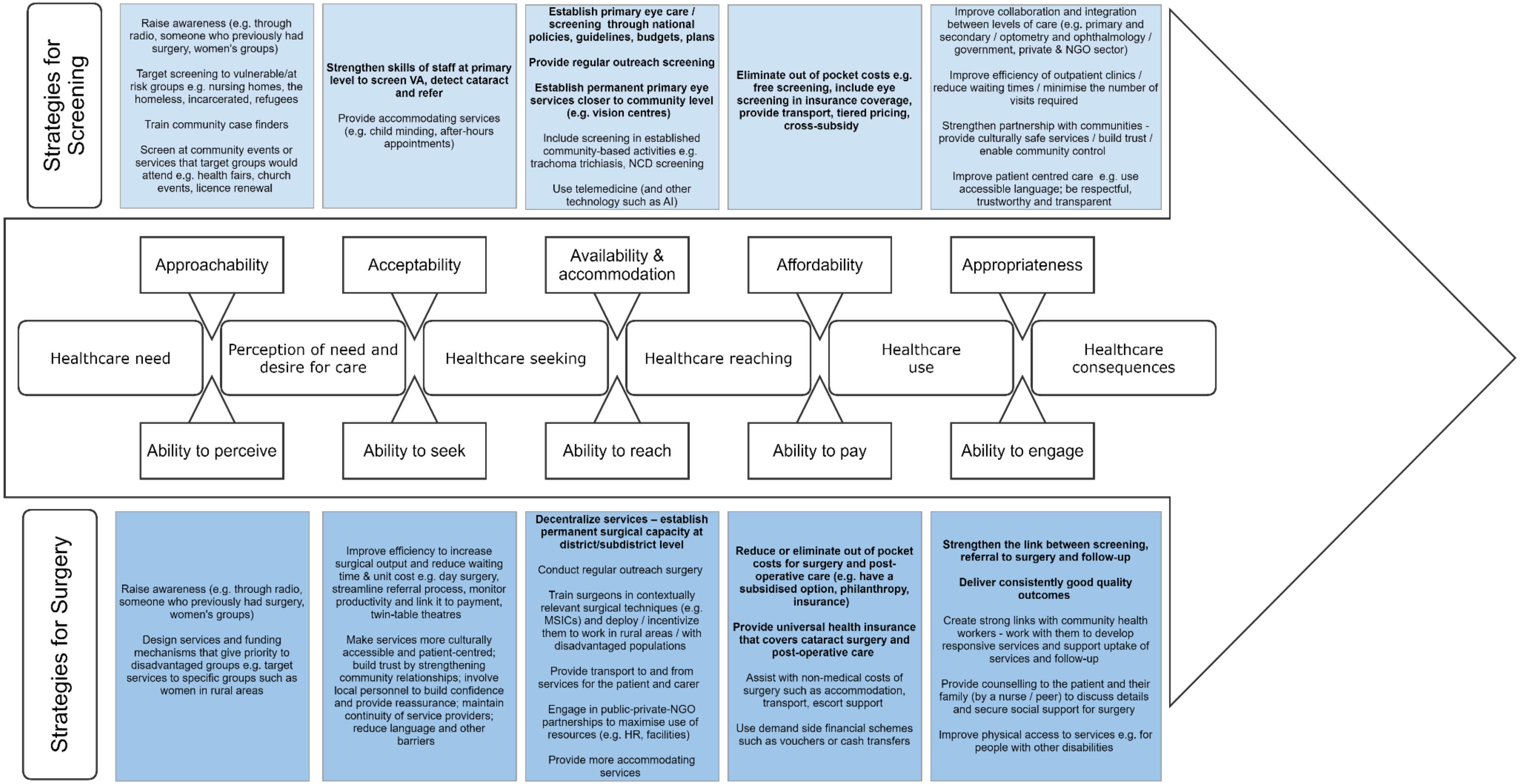

Supplement: S3 Fig — (PDF) [file pgph.0000631.s004.pdf]
