# Supplementary material for: Cataract services for all: Strategies for equitable access from a global modified Delphi process
Source: PLOS Glob Public Health. 2023 Feb 22;3(2):e0000631. doi: 10.1371/journal.pgph.0000631 (PMC10021896; doi:10.1371/journal.pgph.0000631)
Supplement: S1 Acknowledgments — (PDF) [file pgph.0000631.s006.pdf]

S1 Acknowledgements: Collaborators (the Cataract Access Study Group):

Aselia Abdurakhmanova, The Red Crescent Society of Kyrgyzstan, Bishkek, Kyrgyzstan;
Asela P Abeydeera, Association of Community Ophthalmologists of Sri Lanka, Colombo, Sri Lanka;
Ada E Aghaji, University of Nigeria, Enugu, Nigeria;
Lucilla Ah Ching-Sefo, Ministry of Health, Samoa;
Munir Ahmed, Orbis International, Dhaka, Bangladesh;
Doris Alvarado, Comite Vision 2020, Tegucigalpa, Honduras;
Harris M Ansari, Pacific Eye Institute, Suva, Fiji;
María Alejandra Corrales Ávika, Ministry of Health, Costa Rica;
Carlos EL Arieta, Universidade Estadual de Campinas, State Sao Paulo, Brazil;
Simon Arunga, Mbarara University of Science and Technology, Mbarara, Uganda;
Elham Ashrafi, Tehran University of Medical Sciences, Tehran, Iran;
Carla Ayres-Musa, Belize Council for the Visually Impaired, Belize;
Haroon Awan, Avicenna Consulting Associates, Islamabad, Pakistan;
Rafaela S. de Barrios, Universidad de San Carlos de Guatemala, Guatemala;
Muhammed Batur, Yuzuncu Yil University, Van, Turkey;
João Barbosa-Breda, University of Porto, Porto, Portugal;
Nigel H Barker, Queen Elizabeth Hospital, Bridgetown, Barbados;
Fernando Barria vonBischhoffshausen, Hospital Regional de concepcion, Chile;
Joanna M. Black, University of Auckland, Auckland, New Zealand ;
Sandra S. Block, Illinois College of Optometry, Chicago, USA;
Mukharram M. Bikbov, Ufa Eye Research Institute, Ufa, Russia;
Anthea Burnett, University of New South Wales, Sydney, Australia;
Cagatay Caglar, Hitit University, Çorum, Turkey;
Anasaini T Cama, The Fred Hollows Foundation, Melbourne, Australia;
Francisco Contreras Campos, Clinica Ricardo Palma, Lima, Peru;
Miriam R. Cano, Ministry of Health and Social Welfare, Asunción, Paraguay;
Ching-Yu Cheng, Duke-NUS Medical School, Singapore, Singapore;
Ima P. Chima, Independent Public Health Ophthalmologist, Abuja, Nigeria;
Felipe A Chiriboga, Fundacion Oftalmologica Del Valle, Quito, Ecuador;
Sophavid Choum-Starkey, Cambodia;
Chimgee Chuluunkhuu, Orbis Mongolia, Ulaanbaatar, Mongolia;
Jaime A Claramunt, Oftalmontt, Puerto Montt, Chile;
Adrienne Csutak, University of Pécs Medical School, Pécs, Hungary;
David Dahdal, St. John Eye Hospital Group, Jerusalem, Israel;
Alastair K Denniston, University Hospitals Birmingham NHSFT, United Kingdom;
Parami Dhakhwa, Seva Nepal, Kathmandu Nepal;
Rainald Duerksen, CBM, Santa Cruz de la Sierra, Bolivia;
Mona Duggal, Post Graduate Institute of Medical Education and Research, Chandigarh, India;
Islam Elbeih, National Eye Center, Cairo, Egypt;
Epee Emilienne, University of Yaounde, Yaounde, Cameroon;
Gamal Ezz Elarab, Magrabi Foundation, Cairo, Egypt;
Ido Didi Fabian, Tel-Aviv University, Tel-Aviv, Israel;
John Farmer, PNG Eye Care, Papua New Guinea;
Eleonora Favuzza, University of Florence, Florence, Italy;
João M. Furtado, University of São Paulo, São Paulo, Brazil;
Heidy García O, Ministerio de Salud y Protección Social, Bogotá, Colombia;
Theresa Gende, The Fred Hollows Foundation New Zealand, Madang, Papua New Guinea;
Tatiana Ghidirimschi, State University of Medicine and Pharmacy, Chisinau, Republic of Moldova;
Suzanne S Gilbert, Seva Foundation, Berkeley California, USA;
Pedro A. Gómez-Bastar, Instituto de la Visión La Carlota, Montemorelos, Mexico;
Andrzej Grzybowski, University of Warmia and Mazury, Olsztyn, Poland;
Reeta Gurung, Tilganga Institute of Ophthalmology, Kathmandu, Nepal;
Aldiana Halim, Cicendo Eye Hospital, Bandung, Indonesia;
Effendy Bin Hashim, Hospital Pulau Pinang, Penang, Malaysia;
Ava Hossain, OSB Eye Hospital, Dhaka, Bangladesh;
Yiwen Huang, The Fred Hollows Foundation, Kunming City, China;
Bayazit Ilhan, Eye Education and Research Hospital, Ankara, Turkey;
Stephanie Jean Jacques, Roseau, Dominica;
Catherine L Jan, Lost Child's Vision Project, Taree, Australia ;
Sanil Joseph, Aravind Eye Care System, Madurai, India;
Jefitha Karimurio, University of Nairobi, Nairobi, Kenya;
Alejandro Vázquez De Kartzow, Hospital Clínico y Centromed, Viña del Mar, Chile;
Amir B Kello, WHO/AFRO ESPEN, Brazzaville, Congo;
Jan E.E. Keunen, Radboud University Mefical Center, Nijmegen, The Netherlands;
Rajiv Khandekar, King Khaled Eye Specialist Hospital, Riyadh, Saudi Arabia;
Rohit C Khanna, L V Prasad Eye Institute, Hyderabad, India;
Moncef Khairallah, Fattouma Bourguiba University Hospital, Monastir, Tunisia;
Dan Kiage, Kisii Eye Hospital, Kisii, Kenya;
Gatera Fiston Kitema, University of Rwanda, Kigali, Rwanda;

Fatima Kyari, University of Abuja, Abuja, Nigeria;
Sucheta Kulkarni, H. V. Desai Eye Hospital, Pune, India;
Elizabeth Kurian, Mission for Vision, Mumbai, India;
Subeesh Kuyyadiyil, Sadguru Netra Chikitsalaya, Chitrakoot, India;
Guna Laganovska, Riga Stradins University, Riga, Latvia;
Van C. Lansingh, Help Me See, New York, USA;
Alberto D. Lazo Legua, Asociacion Civil Divino Niño Jesus, Lima, Peru;
Srinivas Marmamula, L V Prasad Eye Institute, Hyderabad, India;
Duke Mataka, Vaiola Eye Clinic, Nuku'alofa, Tonga;
Alexander Páez Matallana, Fundación Visión, Fernando de la Mora, Paraguay;
Elenoa Matoto, CWM Hospital, Suva, Fiji;
Milka M Mafwiri, Muhimbili University of Health and Allied Sciences, Dar Es Salaam, Tanzania;
Amel Meddeb-Ouertani, The North African Center for Sight "NADI AL BASSAR", Tunis, Tunisia;
Juan Francisco Yee Melgar, Visualiza, Guatemala;
Sailesh Kumar Mishra, Nepal Netra Jyoti Sangh, Kathmandu, Nepal;
Seyed Farzad Mohammadi, Tehran University of Medical Sciences, Tehran, Iran;
Manfred Mörchen, CBM, Bensheim, Germany;
Lizette Mowatt, The University of the West Indies, Jamaica;
Caleb Mpyet, University of Jos, Jos, Nigeria;
Brigitte Mueller-Schmid, Swiss Red Cross, Bern, Switzerland;
Desiree C. Murray, The University of the West Indies, Trinidad and Tobago;
Neil Murray, Rotorua Eye Clinic, Rotorua, New Zealand;
Gudlavalleti Venkata S Murthy, International Centre for Eye Health, LSHTM, London, UK;
Grace Chipalo Mutati, University Teaching Hospital Eye Hospital, Lusaka, Zambia;
Qais Nasimee, Afghanistan Society of Ophthalmology, Kabul, Afghanistan;
Varanisese R Naviri, Pacific Eye Institute, Suva, Fiji;
Ming Ni, Kunming, China;
Pinar Aydin O'Dwyer, ANKARA, Turkey;
Koichi Ono, Juntendo University School of Medicine, Tokyo, Japan;
Ala Paduca, Nicolae Testemitanu State University of Medicine and Pharmacy, Republic of Moldova;
J Carlos Pastor, University of Valladolid, Valladolid, Spain;
Anna Palagyi, University of New South Wales, Sydney, Australia;
Suzana Pavljasevic, Eye Policlinic Public Health Center, Tuzla, Bosnia and Herzegovena
Huynh Tan Phuc, The Fred Hollows Foundation, Vietnam;
Prabhath Piyasena, Ministry of Health, Colombo, Sri Lanka;
Mundi Q Qoqonokana, Pacific Eye Institute, Suva, Fiji;
Ilsen Patricia Tárraga Quintela, Fundación Ojos del mundo, La Paz, Bolivia;
Muhammad Babar Qureshi, CBM International, Cambridge, UK;
M Mansur Rabiu, Dubai Health Authority, Dubai, UAE;
Shamanna Bindiganavale Ramaswamy, University of Hyderabad, Telangana, India;
Yvonne Reifler, Eye Care Foundation, Amsterdam, The Netherlands;
Ileana Brea Rodríguez, PAHO, Panama;
Pavel Rozsival, Teaching Hospital, Hradec Kralove, Czech Republic;
Paisan Ruamviboonsuk, Rangsit University, Bangkok, Thailand;
Solange Rios Salomão, University of São Paulo, São Paulo, Brazil;
Sare Safi, Shahid Beheshti University of Medical Sciences, Tehran, Iran;
Mohamad Aziz Salowi, Selayang Hospital, Selangor, Malaysia;
Yuddha D Sapkota, International Agency for Prevention of Blindness, Kathmandu, Nepal;
Quacoe-Wossinu Senanu, IAPB Co-Chair, Lome, Togo;
Rodica Sevciuc, Institute of Emergency Medicine, Chisinau, Republic of Moldova;
Indra P Sharma, JDW National Referral Hospital, Thimphu, Bhutan;
Victoria M Sheffield, International Eye Foundation, USA;
Bernadetha Shilio, Ministry of Health National Eye Care Program, Dodoma, Tanzania;
Murray Smith, Melbourne, Australia
Nigel A. St Rose, Trinidad and Tobago Optometrists Association, Tunapuna, Trinidad and Tobago
Maria Remedios Dominique Mapa Suplido, The Fred Hollows Foundation, Makati, Philippines;
John L Szetu, Fred Hollows Foundation NZ, Honiara, Solomon Islands;
Demissie Tadesse, CBM International, Addis Ababa, Ethiopia;
Sandra L Talero, Instituto Barraquer de América, Bogotá D.C. Colombia;
Rabebe Tekeraoi, Ministry of Health and Medical Services, Kiribati;
Benoit Tousignant, University of Montreal, Montreal, Canada;
Vu Tuan-Anh, Vietnam National Eye Hospital, Hanoi, Vietnam;
Marisela Salas Vargas, Caja Costarricense Seguro Social, San Jose, Costa Rica;
Sara Varughese, Christoffel Blinden Mission, India;
Bruna V. Ventura, Altino Ventura Foundation, Recife, Brazil;
Rodrigo A Vidal, Universidad de Chile, Facultad de Medicina, Santiago, Chile;
Lingam Vijaya, Sankara Nethralaya, Chennai, India;
Gianni Virgili, University of Florence, Italy;
Ningli Wang, Capital Medical University, Beijing, China;
Boateng Wiafe, Operation Eyesight Universal, Accra, Ghana;
Dawn Woo-Lawson, Kingston, Jamaica;
Min Wu, The Affiliated Hospital of Yunnan University, Kunming, China;
Sumrana Yasmin, Sightsavers, Islamabad, Pakistan;
Mariano Yee, Visualiza, Guatemala;
Sangchul Yoon, Yonsei University, Seoul, South Korea;
Mayinuer Yusufu, Capital Medical University, Beijing, China;
Khaidarov Zarif, Avicenna Tajik State Medical University, Dushanbe, Tajikistan
